# Underlying Differences Between Chinese Omnivores and Vegetarians in the Evaluations of Different Dietary Groups

**DOI:** 10.3389/fpsyg.2019.02644

**Published:** 2019-12-06

**Authors:** Qirui Tian, Qingyang Zheng, Shouxin Li

**Affiliations:** School of Psychology, Shandong Normal University, Jinan, China

**Keywords:** attitude, evaluation, omnivore, vegetarian, meat

## Abstract

Drawing upon self-categorization theory, the present research investigated the attitudes of omnivores and vegetarians toward five dietary groups, including omnivores, conscientious omnivores, semi-vegetarians, *vegetarians*, and vegans. When they had high (vs. low) meat rationalization, omnivore participants had fewer negative attitudes toward and more positive evaluations of the omnivore groups but more negative attitudes toward and fewer positive evaluations of the vegetarian groups. Vegetarian participants had the most negative attitudes toward the omnivore group, followed by the conscientious omnivore group, the semi-vegetarian group, the vegetarian group, and the vegan group; the vegetarian participants with high meat rationalization (vs. those with low meat rationalization) had more positive evaluations of the omnivore groups. Such findings suggested that high levels of meat-eating rationalization predicted more favorable attitudes toward omnivores among both omnivore and vegetarian participants.

## Introduction

Humans have sought meat for millennia (Rozin, 2004) because most people consider meat to be the chief source of protein and calories. Fiddes (1991) even suggested the existence of “the Protein Myth,” which directly equates meat to protein. In addition to its nutritional value, meat is also delicious to most people. For example, in an Australian sample, the strongest barriers preventing people from becoming vegetarians were the enjoyment of eating meat and an unwillingness to change this dietary habit (Lea and Worsley, 2003). Even so, vegetarian people, of course, do not eat meat.

Regarding the different dietary types, omnivore and vegetarian are not the only types. Based on different categorical standards, both have different subtypes. According to their meat-eating frequency and type, people can be generally divided into five dietary groups: omnivores, who have no restriction on food consumption and consume animal flesh (meat); conscientious omnivores, who only consume animal flesh that has met certain ethical standards (Singer and Mason, 2006); semi-vegetarians (flexitarians), whose diet is plant-based with the occasional inclusion of animal flesh; *vegetarians*, who refuse to eat all animal flesh; and vegans, who do not consume any animal flesh or animal byproducts, such as milk, eggs, and items refined or manufactured from animals, including animal-tested baking soda or fur clothing. These five dietary groups, while not intended to reflect the entire range of human dietary choices, seem to generally represent a transition of the human diet from omnivore to vegan dietary patterns. To our knowledge, there has been little research that simultaneously includes these five groups and assesses the attitudes of omnivores and vegetarians toward them, and this represents a knowledge gap that our studies aimed to address.

The variety of human dietary types can not only be categorized according to different dietary groups but also affects the positive or negative attitudes of omnivores and vegetarians toward each other. To explore the attitudes of omnivores and vegetarians toward each other would contribute to other recent attempts to understand the basis of differences in attitudes between different dietary groups (e.g., Chin et al., 2002; Ruby and Heine, 2011; Minson and Monin, 2012; Ruby, 2012; Ruby et al., 2016; MacInnis and Hodson, 2017; Judge and Wilson, 2019) and to discuss how attitudes can be used to facilitate relationships between omnivores and vegetarians. With the aim of investigating the differences in attitudes between omnivores and vegetarians, the present research examined omnivores’ attitudes toward vegetarians and vegetarians’ attitudes toward omnivores in two Chinese samples. To do this, we drew upon self-categorization theory, one of the most expansive theories in contemporary social psychology.

### Self-Categorization Theory and Differences in Attitudes Between Omnivores and Vegetarians

Building upon social identity theory (Tajfel, 1982), self-categorization theory posits that individuals categorize themselves with others who have the same characteristics with them in relation to some aspect and view themselves as members of these distinct categories (Turner et al., 1987). Considerable research has shown that people tend to treat ingroup members preferentially and to derogate outgroup members (e.g., Tajfel et al., 1971). As such, self-categorization theory can play a critical role in providing an understanding of the attitudes of those with different dietary identities. Due to different dietary standards or habits, it is very possible that omnivores and vegetarians would easily categorize themselves as belonging to distinct social categories. Such a categorization assigns different statuses to omnivores and vegetarians: omnivores are the majority, and vegetarians are the minority, partially because meat is too palatable for most people to abandon eating it. The majority status of omnivores may make them likely to show negative attitudes toward vegetarians.

Several perspectives provide explanations for the negative attitudes of omnivores toward vegetarians. First, from the perspective of moral comparison, omnivores may anticipate moral reproach from vegetarians because the dietary differences between omnivores and vegetarians might make omnivores believe that vegetarians would consider themselves to be morally superior (Minson and Monin, 2012). Indeed, merely presenting omnivores with a description of vegetarians led to omnivores’ cognitive dissonance about the morality of their own meat-eating behaviors (Rothgerber, 2014a). Second, from the perspective of intergroup relations, vegetarians may represent a symbolic threat to the societal norms of omnivores’ dietary practices because meat-eating is mainstream in the human diet. As such, meat eaters were observed to report more negative attitudes toward vegetarians than to omnivores, and vegetarians themselves also reported negative experiences as a result of their vegetarianism (MacInnis and Hodson, 2017). In addition, a recent study revealed that omnivore participants from New Zealand treated vegetarians as deviant and dissident (Judge and Wilson, 2019). Third, from the perspective of vegaphobia, some omnivores may have a vegaphobic bias. Negative attitudes toward vegans could be a strategy for nonvegans to cope with vegaphobia (Cole and Morgan, 2011). Even in the armed forces, because of the masculine values of power and virility, which can be supported by meat-eating, soldiers also seem to show vegaphobia in that that they were found to regard vegetarians as outsiders and to evaluate vegetarians’ meals as boring, tasteless, and unnourishing (Kildal and Syse, 2017).

On the other hand, the minority status of vegetarians may easily distinguish them from others in terms of food consumption. According to the Unified Model of Vegetarian Identity, vegetarians have developed a special social identity because of their dietary preferences (Rosenfeld and Burrow, 2017). Such a special identity may induce vegetarians to treat others who do not share food-related attributes as threats, and this may activate defensive processes, further leading to negative attitudes toward others (e.g., Branscombe and Wann, 1994); becoming a vegetarian is a symbol of making changes both to one’s personal identity and social identity (Rosenfeld and Burrow, 2018). Although there has been no direct evidence to suggest that vegetarians have negative attitudes toward omnivores, some evidence of negative attitudes between vegetarian subgroups has provided insight for us to predict the negative attitudes of vegetarians toward omnivores. For example, *vegetarians* thought vegans’ diet was restrictive, and vegans described *vegetarians* as hypocritical (Povey et al., 2001). Ethical *vegetarians* perceived health *vegetarians* to be selfish, boring, inferior, and not radical enough (Fox and Ward, 2008), and they also had less favorable evaluations of health *vegetarians* than of vegans (Rothgerber, 2014b). In intragroup evaluations of vegans, they evaluated other vegans who consumed animal flesh more critically than did health and ethical *vegetarians* (Rothgerber, 2014c).

### The Rationalization of Meat-Eating Behaviors

In addition to social categorization, people’s own cognition of meat and meat-eating behaviors also affects their attitudes toward different dietary groups. Most meat eaters definitely like eating meat, and most vegetarians have previously eaten meat in their lives before becoming vegetarians; some vegetarians give up a vegetarian diet and return to eating meat (Beardsworth and Keil, 1991; Menzies and Sheeshka, 2012). The differences between omnivores and vegetarians may reflect their different cognitive rationalizations of meat-eating behaviors. Piazza et al. (2015) introduced 4Ns to summarize people’s rationalization of meat-eating behaviors: natural, normal, necessary, and nice. Specifically, natural refers to the biological basis of meat-eating in human evolution. Normal refers to eating meat as a historically normative behavior for humans. Necessary refers to the essential role of meat-eating in human survival, health, and development. Nice suggests that for most people, meat is palatable and satisfies a human taste need. The 4Ns may reflect common human attitudes toward meat-eating behaviors and meat. A crucial aspect of the present research was to investigate the extent of the rationalization of meat-eating behaviors among Chinese omnivores and vegetarians. It had high relevance to people’s attitudes toward both omnivores and vegetarians and helped reveal the mechanism underlying omnivores’ and vegetarians’ attitudes toward each other. It was expected that when people had high meat rationalization (vs. low meat rationalization), they would have more negative attitudes toward and fewer positive evaluations of vegetarian groups and fewer negative attitudes toward and more positive evaluations of omnivore groups.

## The Present Research

Building on the findings on differences in attitudes between omnivores and vegetarians and self-categorization theory more generally, the present research investigated the hypothesis that Chinese omnivores and vegetarians would have negative attitudes toward each other, which would be moderated by their *rationalization of meat-eating behaviors.* A particularly relevant assumption of self-categorization theory to the current research is that how people self-categorize themselves varied based on the context. Meanwhile, self-categorization theory pays more attention to the multilevel inclusiveness of social categorization (e.g., conscientious omnivore, omnivore, and human) and the multiple categories to which an individual can belong based on different criteria (e.g., a vegetarian, a professor, and a Chinese person) (Ellemers and Haslam, 2011). In any given situation, and based on specific contextual cues, people might flexibly define themselves according to one particular social identity rather than another (Turner et al., 1987). When a social identity is salient because of a particular contextual cue, people’s attitudes and behaviors may be congruent with those that are relevant to the salient identity (Turner, 1999). When presented with diet-related contextual cues, people’s diet-related identities may become salient, and they may then show attitudes and behaviors consistent with the diet-related identity. Therefore, in the present research, it was predicted that omnivore participants would have more negative attitudes toward vegetarian dietary types than toward omnivore dietary types and that vegetarian participants would have more negative attitudes toward omnivore dietary types than to vegetarian dietary types. Furthermore, it was expected that the rationalization of meat-eating behavior would affect people’s attitudes toward omnivores and vegetarians. Study 1 focused on omnivore participants and expected that omnivore participants with high rationalization of meat-eating behaviors would have more negative attitudes toward and fewer positive evaluations of vegetarians than those with low rationalization of meat-eating behaviors and that the opposite would be found with regard to their attitudes toward and evaluations of omnivores. Study 2 focused on vegetarian participants and expected that, when vegetarian participants had high rationalization of meat-eating behaviors, they would have fewer negative attitudes toward and more positive evaluations of omnivores than when they had low rationalization of meat-eating behaviors and that the opposite would be found with regard to their attitudes toward and evaluations of vegetarians.

## Study 1: Omnivores’ Attitudes Toward Different Dietary Groups

### Method

#### Participants

The present research was conducted through an online survey that was distributed with Qualtrics software in China. A total of 494 Chinese individuals who were recruited through a snowball sampling method voluntarily completed the survey. After excluding participants who classified themselves as vegetarians (36), 458 (256 females, 159 males, and 43 missing) participants were retained for the data analysis. The mean age of the sample was 26.04 years (SD_age_ = 10.42). We estimated a medium effect (*f*) of 0.25, which required a total sample of 200 with 80% power to detect any effect in both studies. We exceeded this minimum to ensure that there would be extra participants available to further maximize power and did not conduct analyses until we had finished data collection.

#### Procedure

To investigate the attitudes of Chinese meat eaters toward different dietary groups, we defined five different dietary groups, i.e., omnivores, conscientious omnivores, semi-vegetarians, *vegetarians*, and vegans, in short paragraphs; these definitions were similar to those provided in the beginning of the section “Introduction.” Participants were randomly assigned to one of the five dietary groups. In each group, participants first read the definition of the dietary group and then reported their attitudes toward and evaluations of that dietary group. After the evaluation, participants completed one scale to measure their meat rationalization. Finally, demographic information, such as age, gender, vegetarian or not, and personal dietary habits, such as days of eating meat per month, days of eating meat per week, and amounts of meat eaten per day, were collected. All scales were translated from English to Chinese by two native Chinese speakers. Back translation into English was performed by one bilingual translator, and discrepancies were resolved through discussion between translators.

#### Measures

##### Attitudes Toward the Dietary Groups

We used two methods adapted from Povey et al.’s (2001) research to measure the omnivores’ attitudes toward different dietary groups. One method involved the assessment of negative attitudes with separate adjectives (hereafter referred to as the negative attitude measure), and the other involved the evaluation of a range of attitudes from negative to positive (hereafter referred to as the evaluation measure). In the negative attitude measure, 13 adjectives were chosen from an open-ended investigation of people’s salient beliefs toward four dietary groups to measure participants’ attitudes toward different dietary groups. Specifically, four positive adjectives (healthy, ethical, environmentally friendly, and nutritionally balanced) and nine negative adjectives (cruel, expensive, inhumane, murderous, horrible, boring and bland, hypocritical, extreme, and unnatural) were chosen, and participants were asked whether these adjectives were suitable to depict omnivores, conscientious omnivores, semi-vegetarians, *vegetarians*, and vegans. The participants provided their responses on a seven-point Likert scale (1 = absolutely unsuitable, 7 = absolutely suitable). After reverse-scoring the four positive adjectives, negative attitudes toward the dietary groups were calculated from the mean scores of the 13 adjectives. The Cronbach’s alpha (0.816) showed that the negative attitude measure was reliable.

In the evaluation measure, participants were asked to evaluate how “bad” to “good,” “harmful” to “beneficial,” “unpleasant” to “pleasant,” and “unenjoyable” to “enjoyable” one of the five dietary groups were (Povey et al., 2001). Responses were provided on a 7-point scale from −3 (more negative) to 3 (more positive). Participants’ evaluations of the dietary groups were calculated from the mean scores of the four items. The Cronbach’s alpha (0.889) showed that the evaluation measure was reliable.

##### The 4Ns Scale

The 4Ns scale is a 16-item self-reported scale that measures people’s rationalization of meat consumption (Piazza et al., 2015). The 4Ns are Nature, Necessary, Normal, and Nice, which are measured with four items. Participants indicated to what extent they agreed with the items on a seven-point Likert scale (1 = completely disagree, 7 = completely agree). The Cronbach’s alpha of the 16 items was 0.896 (Natural: 0.689; Necessary: 0.747; Normal: 0.559; Nice: 0.753).

### Results

#### Correlation Analyses

Given the differences among the five dietary groups, correlations between negative attitudes toward and evaluations of the different dietary groups, meat rationalization, and indicators of daily meat-eating frequency were calculated separately for each of the five dietary groups.

As shown in Table 1, when omnivore participants had more negative attitudes toward the five dietary groups, they also showed fewer positive evaluations of each of them. Overall, when omnivore participants ate more meat, as indicated by their meat-eating frequency, they had fewer negative attitudes toward the omnivore and conscientious omnivore groups and more positive evaluations of the omnivore group, whereas they had more negative attitudes toward and fewer positive evaluations of the semi-vegetarian group. Furthermore, omnivore participants had more (fewer) negative attitudes toward and fewer (more) positive evaluations of the vegetarian and vegan groups (the omnivore and conscientious omnivore groups) when they had higher meat rationalization.

**Table 1:** Coefficients of correlations between omnivore participants’ negative attitudes toward and evaluations of the five different dietary groups and meat rationalization as well as indicators of daily meat-eating frequency.

	Groups	Evaluation	DEMM	DEMW	AEMD	MR
Negative attitude	O	−0.679^**^	−0.218^+^	−0.264^*^	−0.185	−0.309^**^
	CO	−0.631^**^	−0.230^*^	−0.132	−0.05	−0.447^**^
	SV	−0.666^**^	0.179	0.250^*^	0.306^*^	0.056
	V	−0.464^**^	0.028	0.031	0.146	0.299^**^
	Vegan	−0.499^**^	0.115	0.102	−0.046	0.440^**^
Evaluation	O		0.397^**^	0.298^**^	0.312^**^	0.375^**^
	CO		0.143	0.126	0.116	0.340^**^
	SV		−0.300^**^	−0.299^**^	−0.277^**^	−0.049
	V		−0.020	−0.028	−0.150	−0.342^**^
	Vegan		0.003	−0.128	−0.158	−0.438^**^

*O = omnivore; CO = conscientious omnivore; SV = semi-vegetarian; V = vegetarian; DEMM = days of eating meat per month; DEMW = days of eating meat per week; AEMD = amounts of eating meat per day; MR = meat rationalization*.

+ p < 0.10;

* p < 0.05;

** *p < 0.01*.

#### Differences in Negative Attitudes Toward Dietary Groups

**Figure 1:**
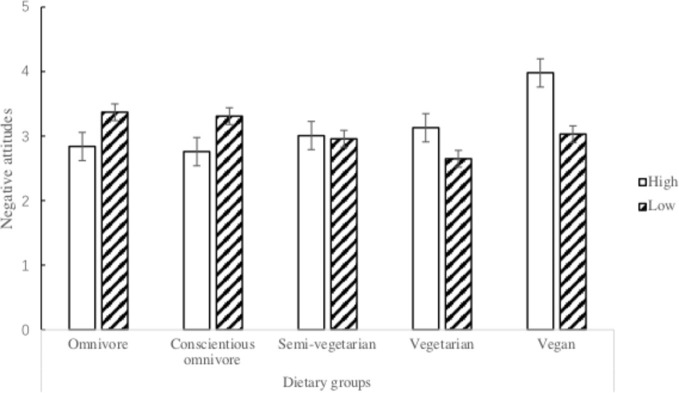
Negative attitudes toward the five dietary groups between omnivore participants with high and low meat rationalization.

A 5 (dietary group: omnivore, conscientious omnivore, semi-vegetarian, vegetarian, and vegan) × 2 (4Ns of meat rationalization: low and high)^2^ between-subject ANOVA was conducted to compare participants’ negative attitudes toward the different dietary groups. The main effect of dietary group was significant, *F*(4, 407) = 3.977, *p* = 0.004, $\eta_{p}^{2}$ = 0.038, but the main effect of the 4Ns of meat rationalization was not significant, *F*(1, 407) = 0.582, *p* = 0.446, $\eta_{p}^{2}$ = 0.001. The main effect of dietary group was further qualified by the significant interaction effect between the dietary group and 4Ns of meat rationalization, *F*(4, 407) = 7.463, *p* < 0.001, $\eta_{p}^{2}$ = 0.068. As illustrated in Figure 1, a simple effect analysis revealed that participants with high meat rationalization had fewer negative attitudes toward the omnivore group, *F*(1, 411) = 5.05, *p* = 0.025, and the conscientious omnivore group, *F*(1, 411) = 5.97, *p* = 0.015, but more negative attitudes toward the vegetarian group, *F*(1, 411) = 3.40, *p* = 0.066, and the vegan group, *F*(1, 411) = 12.99, *p* < 0.001, than those with low meat rationalization. Participants’ attitudes toward the semi-vegetarian group were not different, *F*(1, 411) = 0.08, *p* = 0.783, between those with high and low meat rationalization.

#### Differences in Evaluations of Dietary Groups

**Figure 2:**
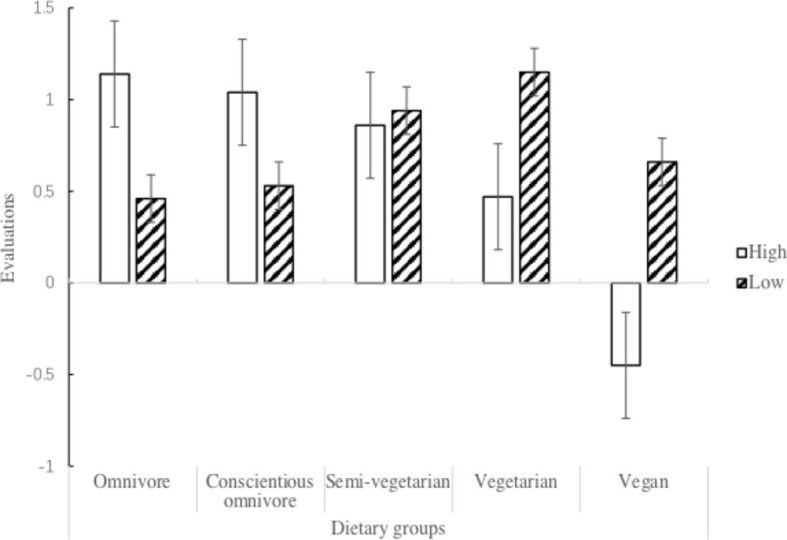
Evaluations of the five dietary groups between omnivore participants with high and low meat rationalization.

A 5 (dietary group: omnivore, conscientious omnivore, semi-vegetarian, vegetarian, and vegan) × 2 (4Ns of meat rationalization: low and high) between-subject ANOVA was conducted to compare participants’ evaluations of the different dietary groups. The main effect of dietary group was significant, *F*(4, 407) = 5.718, *p* < 0.001, $\eta_{p}^{2}$ = 0.053, but the main effect of the 4Ns of meat rationalization was not significant, *F*(1, 407) = 1.322, *p* = 0.251, $\eta_{p}^{2}$ = 0.003. The main effect of dietary group was further qualified by the significant interaction effect between the dietary group and 4Ns of meat rationalization, *F*(4, 407) = 8.256, *p* < 0.001, $\eta_{p}^{2}$ = 0.075. As illustrated in Figure 2, a simple effect analysis revealed that participants with high meat rationalization had more positive evaluations of the omnivore group, *F*(1, 411) = 7.04, *p* = 0.008, and the conscientious omnivore group, *F*(1, 411) = 4.18, *p* = 0.042, than those with low meat rationalization; however, participants with high meat rationalization had fewer positive evaluations of the vegetarian group *F*(1, 411) = 6.63, *p* = 0.01, and even had more negative evaluations of the vegan group, *F*(1, 411) = 13.51, *p* < 0.001, than those with low meat rationalization. There was no difference in the evaluations of the semi-vegetarian group, *p* = 0.709, between participants with high meat rationalization and low meat rationalization.

### Discussion

Study 1 examined the attitudes of omnivore participants toward different dietary groups. As expected, omnivore participants’ negative attitudes toward and evaluations of different dietary groups were dependent on their meat rationalization. When they had high meat rationalization, they had fewer (more) negative attitudes toward the omnivore and conscientious omnivore groups (vegetarian and vegan groups). Regarding evaluations, participants with high meat rationalization evaluated the omnivore and conscientious omnivore groups more positively but evaluated the vegetarian group less positively and the vegan group more negatively. In line with these results, participants’ meat rationalization was negatively correlated with their negative attitudes toward the omnivore groups and with their evaluations of the vegetarian groups but positively correlated with their evaluations of the omnivore groups and with their negative attitudes toward the vegetarian groups. From the perspective of dietary identity (Rosenfeld and Burrow, 2018), one possible explanation was that omnivore participants’ negative attitudes toward vegetarian groups might serve as a way to defend their dietary identities based on meat-eating behaviors. The high meat rationalization of participants might imply that their dietary identities were salient in the diet-related context. A vegetarian diet might threaten their dietary identities. Having more negative attitudes toward and evaluations of vegetarian groups weakened the threat that omnivore participants felt. Such an explanation needs to be accepted with caution and to be examined empirically in future research given that we did not measure the perceived threat that omnivore participants felt.

In addition to omnivores having attitudes toward vegetarians, vegetarians also have attitudes toward omnivores. Further exploring how vegetarians evaluate omnivores could be helpful for us to understand the intergroup attitudes between omnivores and vegetarians. Thus, Study 2 focused on Chinese vegetarian participants to investigate their attitudes toward different dietary groups.

## Study 2: Vegetarians’ Attitudes Toward Different Dietary Groups

### Method

#### Participants

The present study was also conducted online with Qualtrics. To ensure participants were vegetarians as much as possible, the survey was mainly distributed to members of vegetarian associations in China. A total of 356 Chinese who were recruited with the snowball sampling method voluntarily completed the survey. After excluding participants who classified themselves as nonvegetarians in the question about whether they were vegetarians or not (89), 267 (146 females, 62 males, and 59 missing) participants were retained for the data analysis. The mean age of the sample was 29.46 years (SD_age_ = 10.69). A total of 204 participants reported how long they had been vegetarians, and most of them (91.67%) had been vegetarians for a year or more.

#### Measures and Procedure

To investigate the attitudes of Chinese vegetarians toward different dietary groups, the measures and procedure were similar to Study 1. Participants were randomly assigned to read one of the five short paragraphs that described the five different dietary groups, as in Study 1, and then they reported their attitudes toward and evaluations of the dietary groups. After the evaluation, participants completed the meat rationalization scale. Finally, demographic information, such as age, gender, and vegetarian or not, and personal dietary habits, such as days of eating meat per month, days of eating meat per week, and amounts of meat eaten per day, were collected. In addition, the participants’ motivations to be vegetarians, including delicacy, personal health, ethical concerns, environmental protection, weight loss, animal welfare, and religion (Fox and Ward, 2008; Ruby, 2012; Rosenfeld, 2018), were investigated on a seven-point Likert scale.

### Results

#### Correlation Analyses

Correlations of vegetarian participants’ negative attitudes toward and evaluations of the five dietary groups, meat rationalization, and their motivations to be vegetarians were calculated separately for each of the five dietary groups. Motivations to be vegetarians were classified into two types: personal motivation, including delicacy, personal health, and weight loss, and ethical motivation, including ethical concerns, environmental protection, animal welfare, and religion.

As shown in Table 2, when vegetarian participants had more negative attitudes toward the five dietary groups, they also showed fewer positive evaluations of each of them. Vegetarian participants’ negative attitudes toward the five dietary groups were not significantly correlated with their motivations to be vegetarians or their meat rationalization. When vegetarian participants had more ethical motivations to be vegetarians, they had fewer positive evaluations of the omnivore and conscientious omnivore groups. However, when they had higher meat rationalization, they had more positive evaluations of the omnivore and conscientious omnivore groups and fewer positive evaluations of the vegan group.

**Table 2:** Coefficients of correlations between vegetarian participants’ negative attitudes toward and evaluations of the five different dietary groups and meat rationalization as well as motivations to be vegetarians.

	Groups	Evaluation	PM	EM	MR
Negative attitude	O	−0.402^**^	−0.009	0.199	−0.232
	CO	−0.269	−0.127	−0.060	−0.097
	SV	−0.439^**^	−0.093	−0.021	−0.181
	V	−0.310^*^	0.091	0.113	0.017
	Vegan	−0.311^*^	0.076	−0.036	0.177
Evaluation	O		−0.101	−0.409^**^	0.698^**^
	CO		0.065	−0.427^*^	0.435^*^
	SV		0.073	−0.024	0.072
	V		0.055	0.081	−0.210
	Vegan		0.038	0.121	−0.504^**^

*O = omnivore; CO = conscientious omnivore; SV = semi-vegetarian; V = vegetarian; PM = personal motivations; EM = ethical motivations; MR = meat rationalization*.

* p < 0.05;

** *p < 0.01*.

#### Differences in Negative Attitudes Toward Dietary Groups

A 5 (dietary group: omnivore, conscientious omnivore, semi-vegetarian, vegetarian, and vegan) × 2 (4Ns of meat rationalization: low and high) between-subject ANOVA was conducted to compare participants’ negative attitudes toward the different dietary groups. The main effect of the dietary group was significant, *F*(4, 198) = 39.39, *p* < 0.001, $\eta_{p}^{2}$ = 0.443, but the main effect of meat rationalization and its interaction with the dietary group were not significant, *p’*s > 0.29. A Tukey HSD *post hoc* test found that vegetarian participants had significantly more negative attitudes toward the omnivore group (*M* = 4.36, SD = 0.92), conscientious omnivore group (*M* = 3.72, SD = 0.85), and semi-vegetarian group (*M* = 3.20, SD = 0.84) than toward the vegetarian group (*M* = 2.68, SD = 0.72) and vegan group (*M* = 2.52, SD = 0.59) and that vegetarian participants’ attitudes toward the omnivore, conscientious omnivore, and semi-vegetarian groups were also significantly different from each other. The negative attitudes toward vegetarian and vegan groups were not different from each other.

#### Differences in Vegetarians’ Evaluations of Dietary Groups

**Figure 3:**
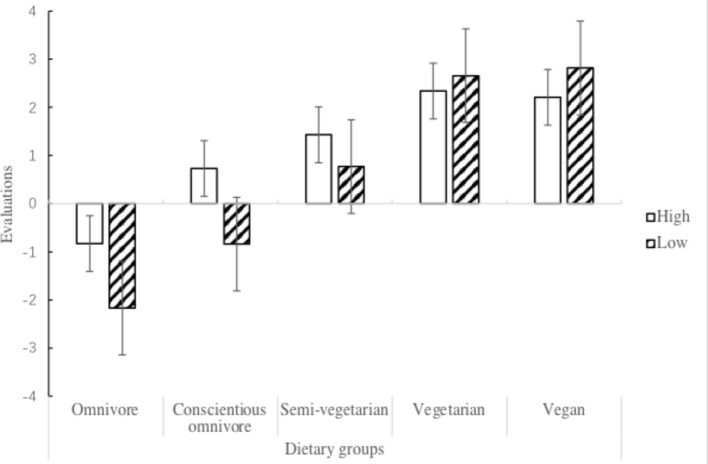
Evaluations of the five dietary groups between vegetarian participants with high and low meat rationalization.

A 5 (dietary group: omnivore, conscientious omnivore, semi-vegetarian, vegetarian, and vegan) × 2 (4Ns of meat rationalization: low and high) between-subject ANOVA was conducted to compare participants’ evaluations of the different dietary groups. The main effect of the dietary group was significant, *F*(4, 203) = 110.51, *p* < 0.001, $\eta_{p}^{2}$ = 0.685, and the main effect of 4Ns of meat rationalization was also significant, *F*(1, 203) = 11.735, *p* = 0.001, $\eta_{p}^{2}$ = 0.055, and this was further qualified by the significant interaction effect between the dietary group and 4Ns of meat rationalization, *F*(4, 203) = 7.963, *p* < 0.001, $\eta_{p}^{2}$ = 0.136. As illustrated in Figure 3, a simple effect analysis revealed that participants with high meat rationalization had fewer negative evaluations of the omnivore group, *F*(1, 207) = 9.61, *p* = 0.002, and more positive evaluations of the conscientious omnivore group, *F*(1, 207) = 6.21, *p* = 0.013, than participants with low meat rationalization. However, there was no difference in the evaluations of the semi-vegetarian group, *p* = 0.283, vegetarian group, *p* = 0.186, and vegan group, *p* = 0.347, between participants with high meat rationalization and low meat rationalization.

### Discussion

Study 2 examined the attitudes of vegetarian participants toward different dietary groups. Vegetarian participants showed the most negative attitudes toward the omnivore group, followed by the conscientious omnivore group, semi-vegetarian group, vegetarian group, and the vegan group based on their vegetarian identities. Their negative attitudes toward different dietary groups were not affected by their meat rationalization, which was also not significantly correlated with their negative attitudes. Because all of the participants in this study were vegetarians, their meat rationalization was very low (*M* = 2.10, SD = 0.78), especially compared with that in Study 1 (*M* = 5.03, SD = 0.97). The low meat rationalization might have had a few effects on the vegetarian participants’ negative attitudes toward omnivore groups. However, when evaluating the different dietary groups, vegetarian participants’ meat rationalization showed that there was an effect on their evaluations of the two omnivore groups. That is, participants with high meat rationalization had fewer negative evaluations of the omnivore group and more positive evaluations of the conscientious omnivore group than those with low meat rationalization. In line with these results, participants’ meat rationalization had significantly positive correlations with their evaluations of omnivores, whereas it had a significantly negative correlation with their evaluations of the vegan group. In the evaluation measure, evaluations ranged from negative to positive for each item. When both negative and positive evaluations were included simultaneously, the relatively high meat rationalization of vegetarian participants could reduce their negative evaluations of the omnivore groups. As the conscientious omnivores had ethical standards regarding meat-eating behaviors, which were somewhat related to concerns for animal welfare, conscientious omnivores were positively evaluated by the vegetarian participants with high meat rationalization. Regarding the vegetarian groups, meat rationalization had no effect. Such results suggested that vegetarian participants, who were very likely to have once been meat eaters, might have fewer negative evaluations of omnivore groups if they do not think eating meat is completely unjustified.

## General Discussion

The current research aimed to understand attitudes toward various dietary groups among Chinese omnivores and vegetarians from the perspective of self-categorization theory. Study 1 revealed that Chinese omnivore participants had more negative attitudes toward and evaluations of vegetarian groups when they had high meat rationalization. Study 2 found that Chinese vegetarian participants reported more negative attitudes toward omnivore groups than toward vegetarian groups. When vegetarian participants had high meat rationalization, they had fewer negative evaluations of omnivore groups.

### Attitudes Toward Vegetarians

Meat eaters usually represent the majority of the population in most countries and play a dominant role in food choices. However, not all meat eaters like eating meat to an equal extent. The effect of meat rationalization on omnivore participants’ attitudes toward and evaluations of different dietary groups indicated that omnivore participants’ attitudes toward and evaluations of different dietary groups were consistent with their rationalization of meat-eating behaviors. That is, when omnivore participants had high (vs. low) meat rationalization, they also had fewer negative attitudes toward and more positive evaluations of omnivore groups and more negative attitudes and fewer positive evaluations of the vegetarian groups. It seemed that omnivore participants’ attitudes toward meat and their own meat-eating behaviors played an important role in affecting their evaluations of vegetarian groups, especially of vegans. For most people, vegetarianism has been viewed as a healthy dietary option, but veganism may still be associated with restriction, deficiency, and extremism (Judge and Wilson, 2015), which may cause vegans to be categorized as a separate group. Furthermore, most people have little knowledge about veganism. When the participants in the vegan group condition learned that vegans did not eat or use anything from animals, they might have thought that the vegan lifestyle was too rigid to allow for any pleasure or that it was impossible to maintain, further leading to the most negative attitudes and the lowest evaluations.

In the current food culture, meat eaters, as the majority, usually take their meat-eating behaviors for granted, whereas vegetarians, as the minority, often represent an identity category that is marked as unique in terms of human food consumption (Rosenfeld and Burrow, 2017). Such differences in group status between omnivores and vegetarians regarding eating behaviors may easily shape omnivores’ attitudes toward vegetarians. For example, when the word “vegan” appeared in newspaper articles in the UK, it was classified as negative in approximately 74% articles (Cole and Morgan, 2011).

### Attitudes Toward Omnivores

The Chinese vegetarian participants’ negative attitudes toward and evaluations of different dietary groups showed a consistent tendency, with the most negative attitudes toward the omnivore group, followed by the conscientious omnivore group, the semi-vegetarian group, the vegetarian group, and then the vegan group, and with the evaluations becoming more positive in the same order. Such tendencies corresponded with the vegetarian identity. Relatively speaking, vegetarians may prefer other vegetarians rather than omnivores who differ from them in terms of food choice.

However, when vegetarian participants thought that eating meat was relatively justifiable, their evaluations became less negative of the omnivore group and more positive of the conscientious omnivore group. The subtypes of vegetarianism imply that not all vegetarians adhere equally strictly to vegetarianism. Most vegetarians are not innate, but they choose to abstain from eating meat at one time in their lives (Beardsworth and Keil, 1991; Hirschler, 2011). With the use of context cues about omnivores, it was possible for vegetarian participants to think more about their past meat-eating behaviors, which led to fewer negative evaluations of the omnivore group and more positive evaluations of the conscientious omnivore group.

### Limitations and Future Directions

Although our research systematically investigated the negative attitudes toward and evaluations of five dietary groups among Chinese omnivore and vegetarian participants, its limitations must be acknowledged.

First, given that vegetarians usually have different motivations to be vegetarians (e.g., Rosenfeld, 2018), one limitation of the current research is that, when defining the five dietary groups, we did not mention or differentiate the motivations of vegetarians. However, the different motivations of vegetarians can often affect people’s evaluations of them. For instance, vegetarians motivated by animal rights were more negatively evaluated by meat eaters than those motivated by personal health or environmental protection (MacInnis and Hodson, 2017). Future research may define vegetarians more specifically and examine meat eaters’ attitudes toward vegetarians with various motivations.

Second, because of the minority status of vegetarians, it was not easy to recruit vegetarian participants in Study 2. Therefore, the inclusion of vegetarian participants in Study 2 lacked specific standards, leading to the possibility that some vegetarian participants might have misrepresented themselves and still eaten meat. Although the placement of the measure of whether participants were vegetarians or not was at the end of the survey and reduced the necessity of participants to misrepresent themselves as vegetarians, about 20% participants among the 204 participants who reported their personal dietary habits admitted that they eat meat 1 day (or some days) in 1 week. Future research would benefit from setting standards to ensure that the targeted participants are truly vegetarians.

Third, it is acknowledged that a direct and specific measure of self-categorization in terms of dietary choices was absent; however, it was expected that the diet-related context of this research could emphasize the corresponding dietary identities of the omnivore participants in Study 1 and the vegetarian participants in Study 2. However, human dietary practices vary with regard to eating meat. For example, when flexitarians self-categorized as vegetarians vs. omnivores on a continuous scale, they categorized themselves as closer to omnivores (Rosenfeld et al., in press). Future research should specifically explore how people’s self-categorization in terms of dietary choices affects attitudes toward different dietary groups.

## Conclusion

In the context of salient dietary identities, both omnivores and vegetarians tended to show negative attitudes toward dietary groups that did not share dietary practices with them. The attitudes of omnivore participants toward different dietary groups depended on their rationalization of meat-eating behaviors. Vegetarian participants also showed more negative attitudes toward omnivores than toward vegetarians, and their rationalization of meat-eating behaviors affected their evaluations of the omnivore groups.

## Data Availability Statement

All datasets generated for this study are available from the Open Science Framework (http://osf.io/pfwna/?view_only=d0595b828e554006bee36a66df60892c).

## Ethics Statement

The studies involving human participants were reviewed and approved by the Human Research Ethics Committee of Shandong Normal University. Written informed consent for participation was not required for this study in accordance with the national legislation and the institutional requirements.

## Authors’ Note

1. With regard to the definition of vegetarianism, there is a lack of consistency, which is a significant challenge for research. In the literature, the term “vegetarian” sometimes includes all types of vegetarians, whereas sometimes it specifies those who do not eat any meat but may eat eggs and dairy products, which is a point of distinction between vegetarian and vegan diets. We used the plural form “vegetarians” to represent the three types of vegetarian diets described in our studies and the single form to represent a specific type of vegetarian. If it is necessary to use the plural form “vegetarians” to represent the specific type of vegetarian, vegetarians would be shown in italics.
2. According to a recent argument about the median split method, it is not problematic to use the median split when the independent variables are not correlated (Iacobucci et al., 2015a,b). The correlations between the five dietary groups (categorical variables) and the 4Ns of meat rationalization (continuous variable) were not significant in Study 1, *ps* = 0.251–0.943, or in Study 2, *ps* = 0.248–0.824, so we used the median split method to divide meat rationalization into high and low levels and compared the differences between participants with the two levels in their attitudes toward and evaluations of the different dietary groups.

## Author Contributions

QT designed the study and wrote the manuscript. QZ designed the study and collected the data. SL provided suggestions on the design and writing. All authors revised and approved the final version of this manuscript.

### Conflict of Interest

The authors declare that the research was conducted in the absence of any commercial or financial relationships that could be construed as a potential conflict of interest.
